# Enhancer RNA-mediated transcriptional regulation of TDP-43 during early neural lineage specification

**DOI:** 10.1080/19768354.2026.2643998

**Published:** 2026-03-26

**Authors:** Yeonju Jang, Hyohi Lee, Myunggeun Oh, Jiin Moon, Seung-Jin Kim, Jaeil Han, Jong-Lyul Park, Seung-Kyoon Kim

**Affiliations:** a Department of Convergent Bioscience and Informatics, and Graduate School of Biological Sciences, Chungnam National University, Daejeon, Republic of Korea; b Department of Biochemistry, College of Natural Sciences, Kangwon National University, Chuncheon, Republic of Korea; c Department of Microbiology and Molecular Biology, Chungnam National University, Daejeon, Republic of Korea; d Personalized Genomic Medicine Research Center, Korea Research Institute of Bioscience & Biotechnology, Daejeon, Republic of Korea

**Keywords:** TDP-43, enhancer RNAs (eRNAs), embryonic stem cells (ESCs), neural lineage specification, epigenetics

## Abstract

TAR DNA-binding protein 43 (TDP-43) is a DNA- and RNA-binding protein that regulates gene expression by modulating transcription and RNA processing. It plays pivotal roles in neuronal development and function, and its mislocalization and aggregation are major pathological features of several neurodegenerative diseases. However, the regulatory mechanisms that control *Tdp-43* expression and activity during the transition from embryonic stem cells (ESCs) to neural progenitor cells (NPCs) remain poorly understood. Through integrative epigenomic and transcriptomic analyses, we identified multiple intergenic and intragenic enhancers within and around the *Tdp-43* locus that generate enhancer RNAs (eRNAs). These eRNAs exhibit dynamic, region-specific expression changes and modulate *Tdp-43* transcription in a stage- and context-dependent manner. Specifically, a subset of eRNAs was highly expressed in ESCs and downregulated upon differentiation, while others were selectively retained or induced in NPCs, paralleling changes in enhancer usage and histone modification states. Targeted knockdown of these eRNAs decreased *Tdp-43* expression and was accompanied by changes in the expression of pluripotency- and lineage-associated markers, without implying direct control over full differentiation trajectories. These findings uncover a previously unrecognized aspect of *Tdp-43* transcriptional regulation and highlight the significance of enhancer dynamics in the epigenetic regulation of TDP-43 expression during early lineage specification.

## Introduction

Transactive response (TAR) DNA-binding protein 43 (TDP-43) is a ubiquitously expressed and evolutionarily conserved DNA/RNA-binding protein implicated in RNA transcription, splicing, transport, and stability (Ling et al. 2013; Reddi 2017; Morera et al. 2019; Duan et al. 2022). Originally identified as a transcriptional repressor of HIV-1 TAR DNA (Ou et al. 1995), TDP-43 contains two RNA recognition motifs (RRM1 and RRM2) and a glycine-rich C-terminal domain that mediate RNA binding and protein–protein interactions, respectively (Buratti et al. 2001; Shin and Brangwynne 2017). Under physiological conditions, TDP-43 is predominantly localized in the nucleus but is capable of shuttling between the nucleus and cytoplasm (Buratti and Baralle 2001; Duan et al. 2022). The protein is indispensable for neural and embryonic development, as global knockout of the *Tdp-43* gene leads to embryonic lethality in mice (Sephton et al. 2010; Wu et al. 2010). In neurons, TDP-43 supports synaptic plasticity, axonal transport, and transcriptomic diversity through effects on mRNA stability and alternative splicing (Handley et al. 2017; Paolicelli et al. 2017; Reddi 2017). In addition to these well-established post-transcriptional functions, TDP-43 is also extensively regulated at the post-translational level, including phosphorylation, ubiquitination, and subcellular localization, mechanisms that are particularly relevant in neurodegenerative disease contexts (Dong and Chen 2018; Suk and Rousseaux 2020; De Boer et al. 2021).

Dysregulation of TDP-43 is a hallmark of neurodegenerative diseases such as amyotrophic lateral sclerosis (ALS) and frontotemporal lobar degeneration (FTLD), where cytoplasmic, ubiquitin-positive cytoplasmic inclusions and contributes to neuronal dysfunction (Neumann et al. 2006; Van Langenhove et al. 2012; Ling et al. 2013). Mutations in the *Tdp-43* gene have also been identified as genetic contributors to ALS (Kabashi et al. 2008; Sreedharan et al. 2008). Although much attention has focused on its pathological aggregation, TDP-43’s central roles in gene expression are increasingly recognized. In addition to its RNA-binding activity, TDP-43 can associate with chromatin and enhancer elements, thereby influencing gene regulatory networks during both normal development and disease (E.P. Consortium 2012; Schwartz et al. 2015; Swain et al. 2016; Ozdilek et al. 2017; Morera et al. 2019). Notably, TDP-43 has been reported to modulate transcription at protein-coding genes and repetitive elements such as Alu retrotransposons, underscoring its broad impact on the transcriptome (Morera et al. 2019). A key feature of TDP-43 is its ability to undergo liquid–liquid phase separation (LLPS), a reversible process that facilitates the spatial organization of RNA processing in membraneless compartments (Conicella et al. 2016; Park and Kim 2025). Recent studies have demonstrated that the phase separation and condensate-forming properties of TDP-43 are crucial for its gene regulatory functions. Specifically, the condensation properties of TDP-43 determine its RNA-binding and regulatory repertoire (Hallegger et al. 2021), while phase separation may contribute to the assembly of regulatory complexes at specific genomic loci, thereby with potential consequences for transcriptional regulation (Grese et al. 2021; Lang et al. 2024). Although LLPS supports normal cellular functions, dysregulation of this process can promote pathological aggregation of the low-complexity domain (LCD) of TDP-43, particularly under stress conditions (Pakravan et al. 2021). Thus, LLPS provides a mechanistic link between TDP-43’s regulatory functions and its propensity for toxic aggregation in disease, bridging its roles in both gene expression and proteinopathy.

Enhancers are a class of *cis*-regulatory elements (CREs) that act in concert with promoters to regulate target gene transcription (Bulger and Groudine 2011; Smith and Shilatifard 2014; Joo et al. 2016; Kim et al. 2019; Gorbovytska et al. 2022; Oh et al. 2025). These regions are transcribed into long non-coding RNAs known as enhancer RNAs (eRNAs), whose expression often correlates with the activation of nearby genes (De Santa et al. 2010; Kim et al. 2010). Typically, eRNAs are approximately 1–2 kilobases in length and are closely associated with the transcriptional activation of specific genes (De Santa et al. 2010; Kim et al. 2019; Prudencio et al. 2020). eRNAs exert their regulatory effects by interacting with transcription factors, transcriptional coactivators, and RNA polymerase II (RNA pol II), thereby facilitating the assembly and stabilization of transcriptional machinery at target gene loci (Kim et al. 2010; Mikhaylichenko et al. 2018). Notably, enhancers that give rise to eRNAs can display bidirectional transcriptional activity, reflecting their dynamic roles in transcription regulation. The transcription of eRNAs is also strongly correlated with specific chromatin signatures indicative of active enhancers, particularly histone H3 lysine 27 acetylation (H3K27ac) and histone H3 lysine 4 mono-methylation (H3K4me1). These histone modifications, along with the presence of chromatin-modifying enzymes at enhancer loci, mark regions of transcriptional competence and enhancer activation. Perturbation studies have shown that suppression of eRNA expression leads to a significant reduction in the transcription of associated target genes, supporting a functional role for eRNAs in facilitating gene activation (Kim and Kim 2013; Lee et al. 2013; Ebrahimi et al. 2019; Moon et al. 2025).

Rather than being mere byproducts of enhancer transcription, eRNAs are now widely recognized as functional regulatory molecules in many contexts, with roles described in developmental processes, cell identity maintenance, and RNA stability. Their dynamic and context-specific roles in gene expression programs position them as promising candidates for therapeutic targeting in disease contexts (Arnold et al. 2019; Ye et al. 2020). Enhancers themselves can be classified into intergenic and intragenic types, based on their genomic location relative to coding genes. Intergenic enhancers are located between genes and act over long genomic distances to regulate the expression of one or more distal genes. In contrast, intragenic enhancers reside within gene bodies, typically in intronic or noncoding regions between exons, and modulate the expression of either their host gene or neighboring genes (Wu et al. 2014; Mikhaylichenko et al. 2018). Together, intergenic and intragenic enhancers represent a versatile and integral component of the gene regulatory network, enabling spatial and temporal control of gene expression across diverse cellular contexts (Khoury and Gruss 1983; Smith and Shilatifard 2014).

Given the limited understanding of how *Tdp-43* expression is regulated during early neural development, and the emerging links between eRNAs and transcriptional control, it is imperative to investigate whether *Tdp-43* is subject to regulation by enhancer-derived eRNAs during ESC-to-NPC differentiation. The dynamic landscape of intergenic and intragenic enhancers in pluripotent cells suggests an additional layer of transcriptional control for key developmental regulators such as TDP-43. Although TDP-43 is known to be primarily regulated at the post-translational level through mechanisms such as phosphorylation, ubiquitination, and subcellular localization (Neumann et al. 2006; Dong and Chen 2018), these processes do not preclude additional and complementary transcriptional inputs. Here, we identify for the first time multiple eRNAs within and around the *Tdp-43* locus through an integrative analysis of epigenomic and transcriptomic data, including chromatin immunoprecipitation sequencing (ChIP-seq) and global run-on sequencing (GRO-seq). Although these public datasets originated from different studies, all were derived from the same or highly similar E14TG2a backgrounds, ensuring biological comparability. Expression analysis using both RPKM quantification and RT-qPCR validation revealed that distinct subsets of *Tdp-43* eRNAs display differential temporal expression patterns during ESC-to-NPC differentiation. Specifically, one subset of eRNAs was highly expressed in mESCs and downregulated upon differentiation, whereas another subset was induced during early neural specification. These findings suggest that eRNAs at the *Tdp-43* locus are associated with dynamic enhancer activity during lineage transitions. In selected cases, eRNA knockdown was accompanied by reduced *Tdp-43* transcript levels and marker changes consistent with altered pluripotency and early neural specification. Together, our findings support a model in which enhancer-associated transcription functions as a complementary regulatory layer upstream of post-translational processes, modulating *Tdp-43* expression during early neural lineage specification while avoiding overinterpretation of broader developmental outcomes.

## Materials and methods

### Cell culture

E14TG2a mouse embryonic stem cells (mESCs) were grown on 0.1% gelatin (Sigma-Aldrich, Missouri; G1890)-coated dishes in the following ESC medium: Glasgow’s Minimum Essential Medium (GMEM, Gibco, Massachusetts; 11710035) containing 10% KnockOut™ Serum Replacement (KOSR, Gibco, Massachusetts; 10828028), 1% fetal bovine serum (FBS, Sigma-Aldrich, Missouri; F0392), 0.1 mM 2-mercaptoethanol (Gibco, Massachusetts; 21985023), 0.1 mM non-essential amino acids, 0.1 mM sodium pyruvate, 0.5% penicillin–streptomycin (all from Welgene, South Korea; LS005, LS013, and LS202-02, respectively) and 1,000 U/ml Leukemia inhibitory factor (LIF, Merck, Hesse, Germany; ESG1106). HEK293 T cells were cultured in Dulbecco’s modified Eagle’s medium (DMEM, Hyclone, Washington; D6429) containing 10% FBS and 1% penicillin–streptomycin. All cells were grown at 37°C in a humidified incubator with 5% CO_2._

### Embryoid body (EBs) formation and neural progenitor cells (NPCs) differentiation

EBs were generated by the spontaneous differentiation of ESCs. Briefly, ESCs were trypsinized (Trypsin-EDTA, Welgene, South Korea; LS015-10) and resuspended in non-gelatin-coated petri dishes containing ESC medium without LIF. For NPC differentiation, the formed EBs were collected by precipitation and cultured in ESC medium without LIF, supplemented with 1 μM retinoic acid (RA, Sigma-Aldrich, Missouri; R2625). The culture medium was replaced every two days. All cells were maintained under standard culture conditions at 37°C in a humidified incubator with 5% CO₂. Importantly, all differentiation experiments and functional validations were conducted in mESCs and NPCs derived from the same parental E14TG2a line, ensuring consistency with the mouse strain background of the publicly available genomic datasets.

### ChIP-Seq data processing and visualization

Publicly available ChIP-Seq datasets from mESCs and NPCs (GEO accession numbers: GSM1000126, GSM4050822, GSE206730, GSM6347289, GSM6347290, GSM2417188, GSM6437307, GSM2651156, GSM4885575, GSM4885576, GSM4885567, GSM4885583, GSM4885591, GSE52386) were processed and analyzed as detailed below. The quality of raw sequencing reads was assessed, and adapter sequences were trimmed using Trim Galore (https://github.com/FelixKrueger/TrimGalore) with default parameters. For paired-end ChIP-Seq datasets, the – paired option was used in Trim Galore to ensure proper trimming of both read pairs. Trimmed reads were aligned to the UCSC mouse reference genome (GRCm38/mm10) using Bowtie2 (https://github.com/BenLangmead/bowtie2) with default settings (Langmead and Salzberg 2012). The aligned reads (SAM files) were converted to BAM format using SAMtools (https://github.com/samtools/samtools) (Li et al. 2009). BAM files were then sorted, and duplicates were removed using Sambamba (https://lomereiter.github.io/sambamba/), with indexing performed simultaneously (Tarasov et al. 2015). To facilitate downstream analysis and visualization, tag directories were created from the processed BAM files using the HOMER suite (http://homer.ucsd.edu/homer/). BigWig files were generated at a 1 bp resolution using the makeUCSCfile command in HOMER, referencing the mm10 genome size. These final BigWig files were subsequently uploaded to the UCSC Genome Browser (https://genome.ucsc.edu/) for visualization of ChIP-Seq signals. Details of the deposited dataset, software, and algorithms are shown in Table S1 and S2. In addition, all public datasets used for ChIP-seq and GRO-seq analyses were derived from mESCs and NPCs of the same or highly similar mouse strain backgrounds (e.g. E14Tg2a), as detailed in Table S1, ensuring biological consistency and comparability across epigenomic profiles.

### Identification of intergenic and intragenic enhancer and enhancer ratio analysis

H3K27ac ChIP-seq datasets (GSM1000126; two replicates) and their corresponding input controls (GEO accession numbers: GSM2308460 and GSM2308461) were used to identify enhancer regions. Sequencing reads were aligned to the UCSC mm 10 mouse reference genome using Bowtie2. After alignment, mapped reads from the two biological replicates were merged using SAMtools to improve robustness prior to enhancer identification. Broad peak calling was conducted with MACS3 (https://macs3-project.github.io/MACS/) using the – broad option to identify regions with wide enrichment of H3K27ac. The resulting peak file was converted to a custom BED format for further analysis. Transcription start sites (TSSs) were identified based on transcript features from the annotated refGene GTF file (v. mm10). A 500 bP flanking window around each TSS (considered as promoters) was excluded from the analysis. The remaining regions were eompiled as the final set of putative enhancers (Gorbovytska et al. 2022). Enhancer regions were further classified based on their overlap with genic regions. Regions overlapping gene bodies were defined as intragenic enhancers, while those located outside gene bodies were defined as intergenic enhancers. This process resulted in two distinct sets of putative enhancers: intragenic enhancers (located within genes) and intergenic enhancers (located outside of genes). In addition, all downstream functional validation experiments (e.g. shRNA knockdown) were conducted in mESCs and NPCs derived from the same parental E14TG2a line, ensuring biological equivalence with the source of genomic datasets.

### The reads per kilobase of transcript per million mapped reads (RPKM) calculation for enhancer and genic regions

Global run-on sequencing (GRO-Seq) reads from mESCs (GEO accession number: GSM2651156) underwent adapter trimming and quality control using Trim Galore with default parameters. Trimmed reads were then aligned to the genome using Bowtie2. Quantification of the total number of aligned reads for both genic and enhancer regions was performed using HTSeq (https://htseq.readthedocs.io/en/latest/). RPKM values for genic regions were calculated based on gene lengths extracted from the ENCODE-annotated GTF file and the total number of aligned reads. Enhancer RPKM values were similarly computed using the previously identified enhancer regions, with peak calling conducted by MACS3. To ensure accuracy, average enhancer RPKM values were calculated after excluding the ±500 bp region surrounding the TSS. RPKM values were determined by normalizing the read counts to the length of the enhancer regions and total number of mapped reads.

### Heatmap analysis

To examine the relationship between H3K27ac peak intensity and GRO-seq read intensity, a heatmap was generated using the deepTools package (https://github.com/deeptools/deepTools). The computeMatrix scale-regions function was employed to construct a matrix based on previously identified enhancer regions (in BED format) and the merged H3K27ac signal (in BigWig format). The enhancers regions were scaled around their peaks to enable comparative analysis. This matrix was then utilized to produce a heatmap with the plotHeatmapfunction. Enhancer peaks were ranked by their H3K27ac intensity, and the corresponding GRO-seq read intensities were visualized in the same hierarchical order to reveal patterns of co-enrichment. For this analysis, three distinct sets of enhancers, derived from the previously identified enhancer regions, were included.

### RNA extraction and cDNA synthesis

RNA was extracted from cells cultured in 6-well cell culture plates using TRIzol reagent (Invitrogen, Massachusetts; 15596018) as previously described (vI. Jung et al. 2012). Briefly, 1 ml of TRIzol was added to each well, and the plates were incubated on a shaker for 10 min at room temperature (RT). Subsequently, 500 μl of chloroform was added to each sample, followed by vigorous shaking, and the tubes were left to stand for 3 min at RT. Samples were then centrifuged at 12,000 rpm for 10 min at 4°C. The aqueous phase was carefully transferred to a new tube containing an equal volume of isopropanol, and the mixture was incubated at – 30°C for 30 min to promote RNA precipitation. Samples were centrifuged again at 12,000 rpm for 10 min at 4°C. The resulting RNA pellet was washed with 75% ethanol, air-dried, and resuspended in RNase-free water. cDNA was synthesized from 2 μg of total RNA using the M-MLV cDNA Synthesis Kit (Enzynomics, South Korea; EZ006S) according to the manufacturer’s instructions. For cDNA synthesis targeting intragenic eRNAs, random hexamer primers were primarily used.

### Real-time quantitative polymerase chain reaction (RT-qPCR)

RT-qPCR was conducted as previously described (Kim et al. 2021). Briefly, DNA amplification was carried out in a 10 μl reaction mixture containing 15 ng of cDNA, 5 μl of Power SYBR-Green PCR master mix (Applied Biosystems, Massachusetts; 4368708), 5 pmol of each primer, and distilled water. Reactions were carried out on a QuanStudio1 Real-Time PCR system (Applied Biosystems, Massachusetts, A40425) with the following cycling conditions: an initial activation and denaturation step at 50°C for 2 min and 95°C for 10 min, followed by 40 cycles of denaturation at 95°C for 15 s, and annealing, extension, and fluorescence measurement at 60°C for 1 min. All data were analyzed using the ΔΔCT method, with relative mRNA levels normalized to *Tbp*. For the quantification of intragenic eRNA expression levels, normalization was performed by dividing the values obtained from the cDNA synthesized using random hexamers by those obtained from the cDNA synthesized using oligo(dT) primers. The primer sequences are shown in Table S4.

### Short hairpin RNA (shRNA) knockdown

shRNAs against *Tdp-43* were designed as previously described (Kim et al. 2014; Kim et al. 2021). For targeting the *Tdp-43* intergenic eRNA, *Tdp-43* intragenic eRNA, shRNA sequences were generated using the Vectorbuilder design tool. Individual shRNAs were subcloned into the HpaI/XhoI sites of the pLLX lentiviral vector (Zhou et al. 2006). One day prior to transfection, HEK293 T cells were seeded into 100 mm culture dishes. A transfection mixture containing 4 μg of vesicular stomatitis virus (VSV-G) envelope plasmid, 4 μg of delta 8.9 (Δ8.9) plasmid, and 4 μg of shRNA plasmid was prepared. This mixture was combined with polyethylenimine (PEI; 1 mg/ml; Polysciences, Warrington; 23966) in DMEM, incubated for approximately 15 min, and then gently added dropwise to the HEK293 T cell culture medium. The following day, the medium was replaced with a 1:1 mixture of HEK293 T and ESC medium. Four days after transfection, the supernatant containing lentivirus was collected and filtered through a 0.45μm PES syringe filter (0.45 μm; Millipore, Massachusetts; SLHV033RS) to collect the lentivirus. For ESC infection, 500 μl of shRNA lentivirus was added to ESCs cultured in 6-well plates on the same day. The medium was completely replaced the day after infection, and on the third day post-infection, half of the medium was replaced. ESC samples were harvested on the fourth day post-infection for subsequent analysis. For NPCs infection, 500 μl of shRNA lentivirus was added to ESCs in 6-well plates one day prior to infection. The medium was completely replaced the next day. On the second day post-infection, cells were harvested and resuspended in non-gelatin-coated culture dishes containing ESC medium without LIF. On the second day of EB formation, the medium was supplemented with 1 µM retinoic acid (RA, Sigma-Aldrich, Missouri; R2625), and the culture medium was replaced every two days. NPCs samples were harvested on the sixth day of differentiation for subsequent analysis. The shRNA sequences are shown in Table S5.

### Alkaline phosphatase (AP) staining

AP staining was conducted as previously described (Kim et al. 2014). Briefly, four days after lentiviral infection, the cells were fixed with 4% paraformaldehyde solution for 2 min at RT and then washed with PBS-T (PBS containing 0.1% Tween-20). Subsequently, the cells were incubated with an AP staining solution prepared by combining 1 mg/mL Fast Red Violet LB salt solution (Sigma-Aldrich, Missouri; F3381-100MG), 4 mg/mL Naphthol AS-BI phosphate solution (Sigma-Aldrich, Missouri; N2125-500MG), and 2 mol/L AMPD buffer (Sigma-Aldrich, Missouri; A9754-25G) in distilled water. Cells were incubated with the staining solution in the dark at RT for 15 min, followed by washing with PBS-T to remove excess stain. Images were obtained using a microscope.

### Immunofluorescence analysis

Immunofluorescence analysis was conducted as previously described (Kim et al. 2014) with minor modifications. We prioritized immunofluorescence to retain spatial resolution and capture cell-to-cell variability during early neural specification, particularly for proteins with heterogeneous expression or localization such as TDP-43 and NESTIN. Western blotting was subsequently performed as a complementary bulk validation where appropriate. Cells were fixed with 3.7% paraformaldehyde (prepared by diluting 37% paraformaldehyde in distilled water; Sigma-Aldrich, Missouri; 252549-100ML) at 4°C in the dark for 30 min, followed by three washes with PBS-T (5 min each). Permeabilization was carried out using 0.1% Triton X-100 (diluted in distilled water; LPS Solution, South Korea; TRX01) for 20 min with gentle shaking, followed by three additional washes with PBS-T. Blocking was performed with 3% bovine serum albumin (LPS Solution, South Korea; BSA100) at room temperature for 1 h. Cells were then incubated with primary antibodies against TDP-43 and NESTIN (Cell Signaling Technology, Massachusetts; 89789S and 10959, respectively) for 2 h at room temperature, followed by six washes with PBS-T. Alexa Fluor 488-conjugated secondary antibodies (for TDP-43, diluted 1:300 in PBS-T; Invitrogen, Massachusetts; A-11018) and 594-conjugated secondary antibodies (for NESTIN, diluted 1:300 in PBS-T; Invitrogen, Massachusetts; A-11012) were applied for 1 h at room temperature in the dark, followed by six additional PBS-T washes. Nuclei were counterstained with DAPI (4′,6-diamidino-2-phenylindole; Abcam, Cambridge; ab104139), and images were acquired using a fluorescence microscope.

Fluorescence quantification was carried out by manually selecting regions of interest (ROIs) corresponding to individual cells in ImageJ/FIJI. For each ROI, mean fluorescence intensity per cell was measured without subcellular segmentation. Background subtraction and DAPI-based normalization were not applied, as fluorescence intensities were consistently above background and nuclear DNA content was not directly relevant to protein quantification. To minimize sampling bias, imaging fields with comparable cell density were chosen across conditions. For NESTIN, quantification was restricted to cells with clearly delineated cytoplasmic filament structures to reduce variability. All images were acquired under identical exposure and gain settings across conditions. Quantification yielded consistent results across biological replicates.

### Western blot analysis

Western blot analysis was carried out as previously described with minor modifications (Halanobis et al. 2025). Cells cultured in 6-well plates were washed twice with ice-cold PBS and lysed on ice with 100 µl RIPA buffer (Biosesang, South Korea; R2002) per well supplemented with 1× protease inhibitor cocktail (Gendepot, Texas; P3100), followed by a 10-min incubation. Lysates were scraped, transferred to microcentrifuge tubes, and further incubated on ice for 10 min with intermittent vortexing. Samples were adjusted to 1× Laemmli sample buffer (62.5 mM Tris-HCl [pH 6.8], 2% SDS, 10% glycerol, 5% β-mercaptoethanol, and 0.01% bromophenol blue), briefly sonicated, heated at 95°C for 5 min, and clarified by centrifugation at 16,000 × g for 15 min at 4°C. Supernatants were used immediately or stored at – 80°C. Equal volumes (15 µl per lane) were loaded together with a protein ladder (GeneStar, South Korea; GPM2700) onto 4% stacking gels over 10% resolving gels. Gels were pre-run at 60 V for 5 min, followed by electrophoresis at 60 V for ∼25 min through the stacking gel and 120 V for ∼60-90 min through the resolving gel. Proteins were transferred to Nitrocellulose membranes using a wet-transfer system at 300 mA for 2.5 h at 4°C in Tris-glycine buffer with 20% methanol (0-0.05% SDS added where indicated). Transfer efficiency and equal loading were verified by Ponceau S staining (Sigma-Aldrich, Missouri; P3504-10G) prior to blocking. Membranes were washed three times in TBS-T (20 mM Tris-HCl [pH 7.4-7.6], 150 mM NaCl, 0.1% Tween-20), blocked with 5% skim milk in TBS-T at room temperature for 1.5 h, and washed again three times. Primary antibodies against TDP-43, NANOG and NESTIN (Cell Signaling Technology, Massachusetts; 89789S, 8822T and 10959, respectively) (1:1,000 in blocking buffer) were applied for 2 h at room temperature or overnight at 4°C, followed by three washes in TBS-T. HRP-conjugated secondary antibodies (1:5,000 in TBS-T) were then applied for 2 h at room temperature, followed by four washes. Signals were developed using ECL substrate and imaged with a chemiluminescence detection system. Band intensities were quantified using ImageJ/FIJI and normalized to α-Tubulin (Cell Signaling Technology, Massachusetts; 2144S), with Ponceau S profiles additionally used to verify uniform loading and transfer.

### eRNA rescue assay

eRNA rescue assay was carried out as previously described with minor modifications (Moon et al. 2025). To evaluate the functional rescue capacity of the intragenic eRNA, we generated a lentiviral expression vector encoding the wild-type (WT) 123-nucleotide (nt) eRNA sequence. The WT eRNA sequence was cloned downstream of the U6 promoter in the pLLX lentiviral vector using the same cloning strategy employed for shRNA constructs. Vector design was informed by previous studies highlighting the functional importance of the 5′ regions of lncRNAs (Aw et al. 2016; Mustoe et al. 2018; Gorbovytska et al. 2022). Lentivirus production was carried out as described in the shRNA knockdown section. To assess the effect of WT eRNA overexpression alone, control ESCs were independently infected with the WT eRNA lentivirus on day 1 without prior shRNA treatment and harvested on day 4 for comparison. For rescue experiments, ESCs were first transduced with 500 μl of shRNA-expressing lentivirus on day 0. On day 1, cells were reinfected with 1,500 μl of lentivirus encoding the WT eRNA construct. Culture medium was completely replaced on day 2, and cells were harvested on day 4 post-shRNA infection for downstream analyses. Expression of the WT eRNA was verified by RT-qPCR.

### Statistical analysis

All statistical analyses were performed using Microsoft Excel. Unpaired Student's t-tests were applied to calculate *p*-values for RT-qPCR. Data are presented as the mean ± standard error of the mean (SEM), with at least three independent biological replicates included for each condition. Statistical significance was determined using the following thresholds: * *p* < 0.05, ** *p* < 0.01, *** *p* < 0.001, and n.s. (not significant).

## Results

### Epigenomic and transcriptomic profiling identifies intergenic and intragenic enhancers of Tdp-43 in mESCs

**Figure 1.**
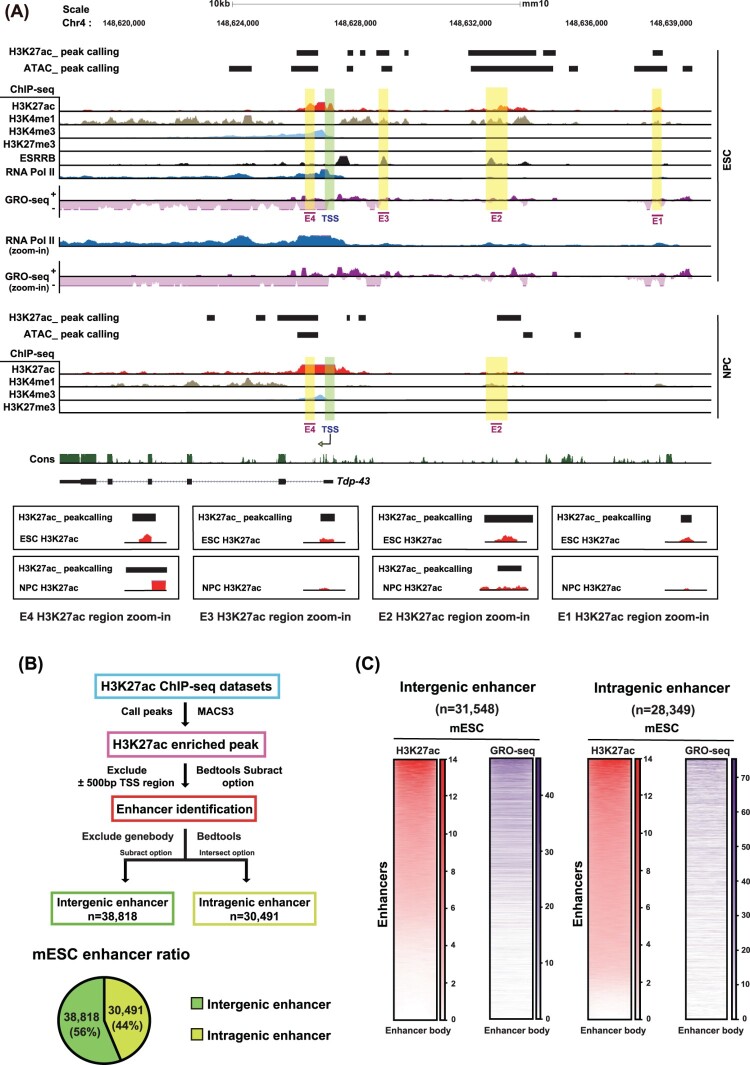
Identification of enhancers of *Tdp-43* in ESCs (A) UCSC Genome Browser view of the *Tdp-43* locus showing ChIP-seq profiles (H3K27ac, H3K4me1, H3K4me3, H3K27me3, ESRRB, and RNA Pol II) along with GRO-seq data in mESCs. GRO-seq tracks show strand-specific nascent transcription, with the top track representing the plus strand and the bottom track representing the minus strand. ‘Cons’ indicates PhastCons conservation scores across multiple vertebrate species based on the mm10 genome alignment. Corresponding ChIP-seq tracks from NPCs are shown for comparison. Putative enhancer regions are highlighted in yellow boxes, and transcription start sites (TSSs) are marked with green boxes. The magnified panels display H3K27ac ChIP-seq signal (red) and peak calling results (black) for ESCs and NPCs at each enhancer region E1-E4. (B) A schematic overview of the workflow used to identify enhancer elements (top). The accompanying pie charts (bottom) illustrate the distribution of intergenic and intragenic enhancers identified in mESCs. (C) Heatmaps displaying H3K27ac ChIP-seq and GRO-seq signal intensities across intergenic (n = 38,818) and intragenic (n = 30,491) enhancer regions in mESCs.

Given the growing recognition of eRNAs in transcriptional regulation (Bulger and Groudine 2011; Smith and Shilatifard 2014; Joo et al. 2016; Gorbovytska et al. 2022), we performed an integrative epigenomic and transcriptomic analysis to investigate the regulatory elements controlling *Tdp-43* expression during early neural differentiation. Our primary goal was to identify both intergenic and intragenic enhancers associated with the *Tdp-43* locus and evaluate their activity in mESCs and NPCs. To this end, we analyzed ChIP-seq datasets for histone modifications indicative of enhancer and promoter activity in both mESCs and NPCs. These included the enhancer marks histone H3 lysine 27 acetylation (H3K27ac) and histone H3 lysine 4 mono-methylation (H3K4me1), the active promoter mark histone H3 lysine 4 tri-methylation (H3K4me3), and the repressive mark histone H3 lysine 27 tri-methylation (H3K27me3) together with strand-specific GRO-seq data. We also included ChIP-seq tracks for RNA polymerase II (RNA Pol II) and ESRRB (Estrogen-Related Receptor Beta), a core pluripotency transcription factor, as well as evolutionary conservation scores (PhastCons) to facilitate enhancer annotation (Figure 1(A)). Candidate enhancers (E1-E4) were identified based on the co-enrichment of H3K27ac and bidirectional nascent transcription detected by strand-specific GRO-seq, as illustrated in Figure 1(B and C). While the publicly available datasets were obtained from different studies, they were derived from matched mESC and NPC samples from the same mouse strain background (Table S1). All functional validations, including knockdown experiments, were performed in ESC-NPC pairs directly derived from the same E14TG2a cell line to ensure consistency between genomic analyses and experimental assays.

A focused analysis of H3K27ac signals revealed several H3K27ac-enriched regions around the *Tdp-43* transcription start site (TSS) in ESCs. To further assess enhancer activity, we integrated these data with nascent RNA sequencing, GRO-seq, which measures nascent transcription. Genome-wide analysis of H3K27ac peaks in mESCs showed that 56% of putative enhancers were intergenic and 44% were intragenic (Figure 1(B), Table S3). Enhancers marked with strong H3K27ac signals exhibited high GRO-seq read density, indicating transcriptionally active enhancers across both intergenic and intragenic regions (Figure 1(B,C), Fig. S1A). Based on co-enrichment of H3K27ac and H3K4me1, along with evidence of bidirectional transcription, which is a hallmark of active enhancers (Lee et al. 2013; Schaukowitch et al. 2014), we identified four candidate enhancer regions, designated E1-E4. Among these, E1-E3 were intergenic, while E4 was located within the *Tdp-43* gene body (intragenic), indicating that active enhancers are distributed both upstream of and within the *Tdp-43* locus (Figure 1(A,B)). Notably, in NPCs, intergenic enhancers E1 and E3 were markedly diminished, while the intragenic enhancer E4 was reinforced (Figure 1(A)). These findings suggest that enhancer-derived eRNAs originating from both intergenic and intragenic regions may cooperatively regulate *Tdp-43* transcription in mESCs and that differential enhancer usage is likely to occur during the transition to the NPC stage. To validate the technical quality of our ChIP-seq datasets, we examined a well-characterized positive control locus. A representative genome browser snapshot at the *Pou5f1* and *Klf4* loci, each of which harbors a known enhancer in ESCs, shows robust enrichment of H3K27ac and RNA polymerase II, confirming the expected distribution of enhancer-associated chromatin features (Figure S1B, S1C). This supports the overall validity of our ChIP-seq signals used to define candidate enhancers near the *Tdp-43* locus.

### Tdp-43 eRNAS are actively transcribed in mESCs and exhibit distinct expression patterns

**Figure 2.**
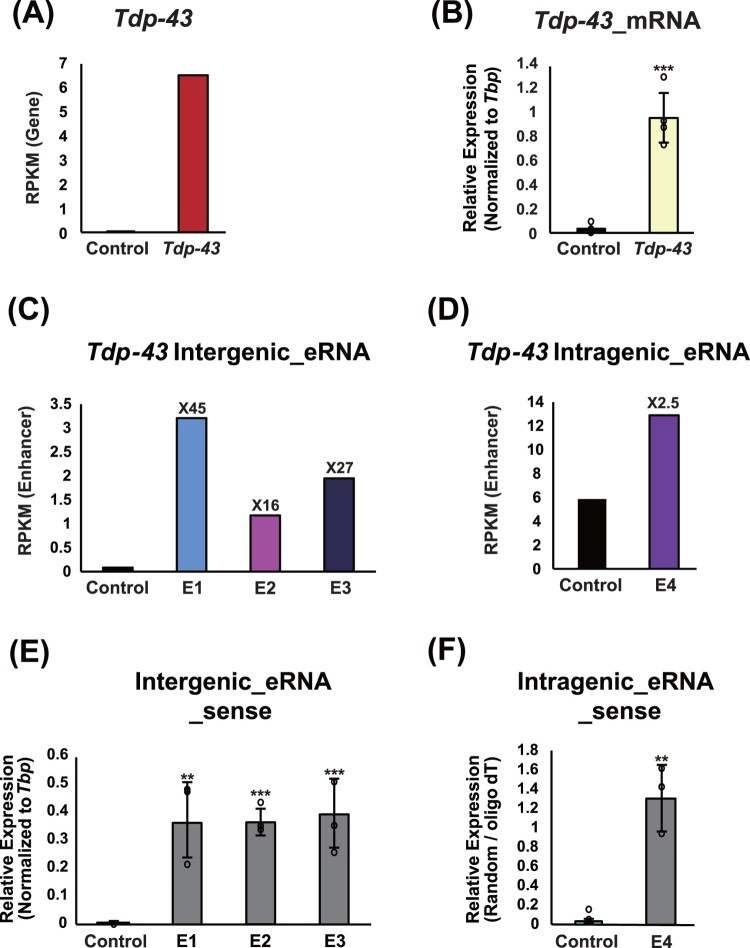
Expression analysis of the *Tdp-43* eRNA and mRNA in mESCs (A) RPKM values comparing *Tdp-43* and the control gene (*T; Brachyury*) in ESCs. (B) RT-qPCR analysis of *Tdp-43* expression relative to the control gene (*T; Brachyury*) in ESCs. RT-qPCR data are presented as mean ± SEM from four independent biological replicates (n = 4). Statistical significance was assessed using an unpaired Student’s t-test. (C, D) RPKM values comparing the *Tdp-43* intergenic enhancers (E1-E3) to the intergenic region of another gene with similar overall expression, *Hirip3* (RPKM = 0.76) (C), and the *Tdp-43* intragenic enhancer (E4) to a non-enhancer intragenic region within the *Tdp-43* gene body (RPKM = 5.39) (D). E1-E4 candidate enhancers were defined using combined H3K27ac and strand-specific GRO-seq signals, representing transcriptionally active regions near the *Tdp-43* locus. (E) RT-qPCR analysis comparing the sense strand expression of *Tdp-43* intergenic enhancers (E1-E3) to the intergenic region of another gene with similar overall expression, *Hirip3* (Control). (F) RT-qPCR analysis comparing the sense strand expression of the *Tdp-43* intragenic enhancer (E4) to a non-enhancer intragenic region within the *Tdp-43* gene body (Control). The expression level of the intragenic eRNA was normalized by dividing the cDNA synthesized with random hexamers by that synthesized with oligo(dT) primers. All RT-qPCR data are presented as mean ± SEM from three independent biological replicates (n = 3), except for Figure 2(B). Statistical significance was assessed using an unpaired Student’s t-test.

To assess the transcriptional activity of putative enhancers at the *Tdp-43* locus, we analyzed GRO-seq data from mESCs. This analysis confirmed robust expression of both *Tdp-43* mRNA and its candidate eRNAs (Figure 2(A, C, and D)), which was further validated by RT-qPCR (Figure 2(B, E, and F)). *Tdp-43* mRNA expression in mESCs was compared to that of *T (Brachyury)*, which exhibits the lowest expression among the tested genes (Figure 2(B)). The expression levels of *Tdp-43* intergenic enhancers (E1-E3) were compared to the intergenic region of a control gene, *Hirip3* (RPKM = 0.76) (2C), which has a similar overall gene expression level to *Tdp-43* (Figure S2A)**.** Additionally, the *Tdp-43* intragenic enhancer (E4) was compared with a non-enhancer intragenic region within the *Tdp-43* gene body (RPKM = 5.39) (Figure 2(D)). In both comparisons, the *Tdp-43* enhancer regions showed significantly higher expression, ranging from approximately 2.5- to 45-fold, indicating strong transcriptional activity at both intergenic and intragenic sites. RT-qPCR further revealed that the sense strands of intergenic eRNAs (E1-E3) were expressed at significantly higher levels than their antisense counterparts in mESCs (Figure 2(E), S2B), consistent with previous findings that synaptic activity preferentially induces sense-strand eRNAs from intergenic enhancers in a strand-specific manner (Schaukowitch et al. 2014). The intragenic eRNA (E4) also showed robust expression in both strands and exceeded expression levels observed at unrelated intragenic regions of the *Tdp-43* gene (Figure 2(F), S2C). Notably, the strand bias of E4 was less pronounced compared to the intergenic eRNAs, suggesting that intragenic enhancers may be governed by a partially distinct transcriptional logic (Figure 2(E,F), S2B, and S2C). Together, these results demonstrate, for the first time, that *Tdp-43* is regulated by both intergenic and intragenic enhancers that are actively transcribed in mESCs, and that their elevated eRNA expression contributes to the precise regulation of *Tdp-43* during stem cell states.

### Tdp-43 enhancer RNAs are required to maintain pluripotency in mESCs by regulating Tdp-43 expression

**Figure 3.**
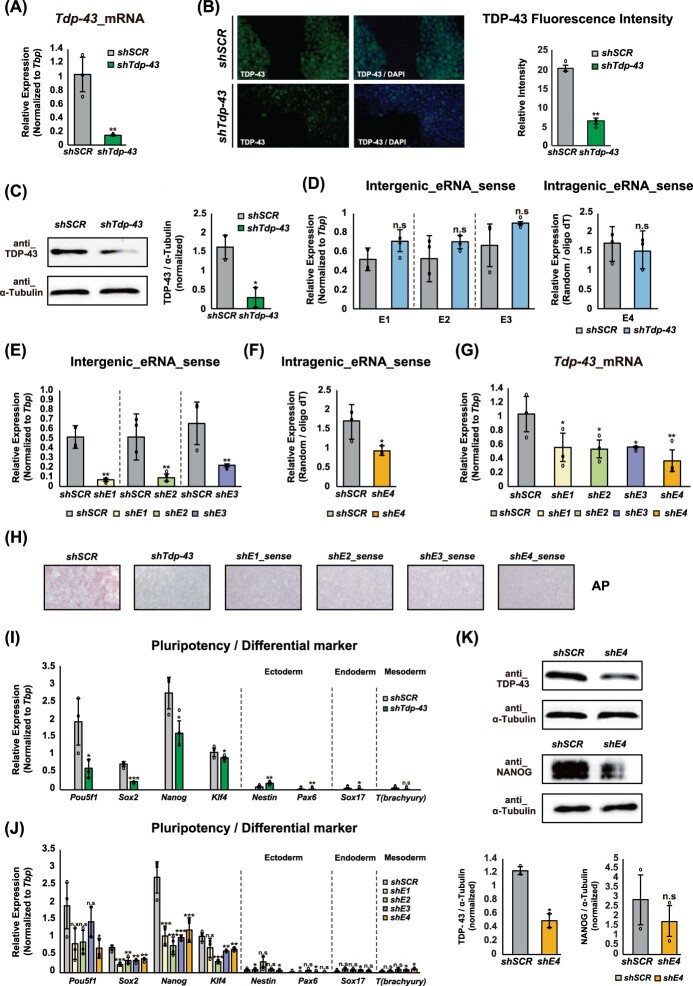
Regulatory roles of *Tdp-43* eRNAs in pluripotency maintenance of mESCs (A) RT-qPCR analysis of *Tdp-43* mRNA expression in mESCs following shRNA-mediated knockdown of either *Tdp-43* or scrambled control. (B) Immunofluorescence analysis of TDP-43 protein expression in mESCs following shRNA-mediated knockdown of *Tdp-43* compared to scrambled control (left). Quantification of fluorescence intensity (right) was performed using ImageJ and is presented as mean ± SEM from two independent biological replicates (n = 2). (C) Western blot analysis of TDP-43 (45 kDa) protein expression in mESCs following shRNA-mediated knockdown of *Tdp-43* compared with scrambled control (left). α-Tubulin (55 kDa) was used as a loading control. Band intensities (right) were quantified using ImageJ and are presented as mean ± SEM from two independent biological replicates (n = 2). (D) RT-qPCR analysis of *Tdp-43* eRNAs (E1-E4, sense strand) following knockdown of *Tdp-43* or scrambled control. (E, F) RT-qPCR analysis of intergenic (E1-E3, E) and intragenic (E4, F) *Tdp-43* eRNA (sense strand) following individual eRNA knockdown compared to scrambled control. (G) RT-qPCR analysis of *Tdp-43* mRNA expression following knockdown of each eRNA compared to the scrambled control. (H) AP staining of mESCs following knockdown of scrambled control, *Tdp-43* mRNA, intergenic eRNAs (E1-E3), or intragenic eRNA (E4, sense strand). (I, J) RT-qPCR analysis of pluripotency markers (*Pou5f1*, *Sox2*, *Nanog*, and *Klf4*) and lineage-specific markers for ectoderm (*Nestin* and *Pax6*), endoderm (*Sox17*), and mesoderm (*T; Brachyury*) following knockdown of scrambled control and *Tdp-43* mRNA (I), or scrambled control, intergenic eRNAs (E1-E3), and intragenic eRNA (E4) (J). The expression level of the intragenic eRNA was normalized by dividing the cDNA synthesized with random hexamers by that synthesized with oligo(dT) primers. All RT-qPCR data are presented as mean ± SEM from three independent biological replicates (n = 3). Statistical significance was assessed using an unpaired Student’s t-test. (K) Western blot analysis of TDP-43 (45 kDa) and NANOG (29-42 kDa) protein levels in mESCs following shRNA-mediated knockdown of the *Tdp-43* intragenic eRNA (E4). α-Tubulin (55 kDa) was used as a loading control. Band intensities were quantified using ImageJ and are presented as mean ± SEM from two independent biological replicates (n = 2).

Given the pronounced changes in enhancer usage observed during the transition from mESCs to NPCs (Figure 1(A)), we sought to define the functional role of *Tdp-43* eRNAs in this context. To this end, we constructed lentiviral vectors encoding small hairpin RNAs (shRNAs) (Schaukowitch et al. 2014) targeting either *Tdp-43* mRNA or individual eRNAs (E1-E4), enabling selective depletion of each transcript. We first introduced shRNAs against *Tdp-43* mRNA and confirmed efficient knockdown, as indicated by a significant reduction in *Tdp-43* expression relative to the scrambled control (Figure 3(A)). This result was further validated by immunofluorescence analysis in mESCs, which showed a marked decrease in TDP-43 protein levels compared to control cells (Figure 3(B)). Consistent with this, Western blotting provided complementary validation, confirming the same reduction in TDP-43 protein expression (Figure 3(C)). Notably, knockdown of *Tdp-43* mRNA did not affect the expression of either intergenic (E1-E3) or intragenic (E4) *Tdp-43* eRNAs, indicating that eRNA transcription occurs independently of *Tdp-43* mRNA expression (Figure 3(D)). Given prior reports that eRNA depletion can downregulate target gene expression (Li et al. 2013; Schaukowitch et al. 2014), we next examined whether *Tdp-43* eRNAs regulate *Tdp-43* transcription. Targeted knockdown of either intergenic or intragenic eRNAs resulted in robust depletion of the corresponding eRNA species relative to the scrambled control (Figure 3(E–G)), and, as expected, was accompanied by a significant reduction in *Tdp-43* mRNA levels (Figure 3(G)). These findings suggest that eRNAs transcribed from both intergenic and intragenic enhancers act locally to promote *Tdp-43* expression in mESCs.

To determine whether this regulation influences pluripotency, we performed alkaline phosphatase (AP) staining and RT-qPCR for core pluripotency and lineage markers. Knockdown of either *Tdp-43* mRNA or its eRNAs led to reduced AP staining intensity, indicating a loss of stem cell identity (Figure 3(H)). We further assessed the expression of core pluripotency markers (*Pou5f1*, *Sox2*, *Nanog, a*nd *Klf4*), and lineage-specific markers for the three germ layers: ectoderm (*Nestin* and *Pax6*), endoderm (*Sox17*), and mesoderm (*T; Brachyury*). Both direct *Tdp-43* knockdown and indirect depletion via eRNA targeting resulted in a significant decrease in the expression of pluripotency markers (Figure 3(I, J)). To further validate this observation at the protein level, we focused on *Tdp-43* intragenic eRNA (E4), which caused the most pronounced reduction in *Tdp-43* mRNA (Figure 3(G)). Western blot analysis confirmed that E4 eRNA knockdown led to a marked decrease in TDP-43 protein levels (Figure 3(K)). In addition, NANOG protein levels were also markedly reduced, likely as a consequence of *Tdp-43* eRNA depletion, indicating a loss of pluripotency. Together, these findings demonstrate that eRNA depletion compromises pluripotency at both the RNA and protein levels.

Interestingly, the reduction in pluripotency gene expression was often more pronounced following eRNA knockdown than with mRNA knockdown, suggesting that *Tdp-43* eRNAs may exert a stronger or more specific regulatory effect on the pluripotency network. By contrast, changes in lineage marker expression were modest, likely due to their low baseline levels in the undifferentiated state. Consistent with a previous study (Schaukowitch et al. 2014), we also observed that antisense strands of eRNAs transcribed from intergenic enhancers were less functionally potent than their sense counterparts (Figure S3A-F). Together, these results demonstrate that *Tdp-43* eRNAs promote *Tdp-43* transcription in mESCs and thereby contribute to the transcriptional network associated with pluripotency. This supports a previous report that TDP-43 supports stem cell identity by stabilizing key pluripotency transcripts such as *Sox2* (Modic et al. 2019). Our findings reveal an enhancer-mediated mechanism through which eRNAs regulate *Tdp-43* expression and sustain the transcriptional network underlying stemness.

### Tdp-43 eRNAs undergo context-dependent regulation during early neural differentiation

**Figure 4.**
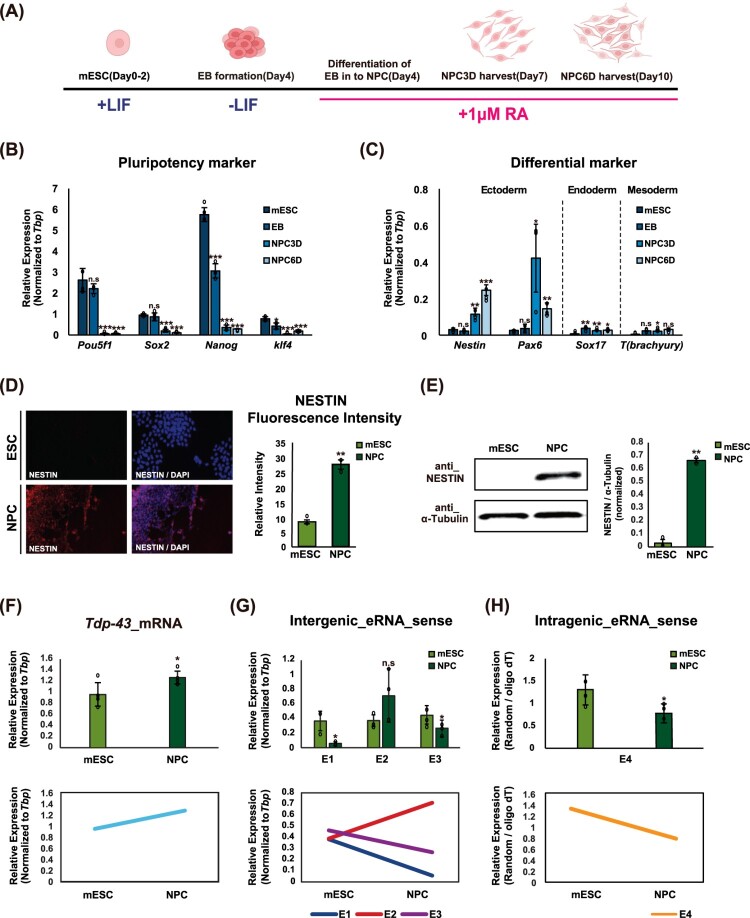
Dynamic expression of *Tdp-43* eRNAs during ESC-to-NPC differentiation (A) Schematic representation of the ESC-to-NPC differentiation protocol used in this study. (B) RT-qPCR analysis of pluripotency markers (*Pou5f1*, *Sox2*, *Nanog*, and *Klf4*) during the differentiation process from ESCs to NPCs. (C) RT-qPCR analysis of lineage-specific differentiation markers for ectoderm (*Nestin* and *Pax6*), endoderm (*Sox17*), and mesoderm (*T;brachyury*) during ESC-to-NPC differentiation. (D) Immunofluorescence analysis of NESTIN protein expression in mESCs and NPCs (left). Quantification of fluorescence intensity (right) was performed using ImageJ and is presented as mean ± SEM from two independent biological replicates (n = 2). (E) Western blot analysis of NESTIN (220 kDa) protein expression in ESCs and NPCs (left). α-Tubulin (55 kDa) was used as a loading control. Band intensities (right) were quantified using ImageJ and are presented as mean ± SEM from two independent biological replicates (n = 2). (F) RT-qPCR analysis of *Tdp-43* mRNA expression in ESC and NPC states, shown as both bar graph (top) and line graph (bottom). RT-qPCR data are presented as mean ± SEM from four independent biological replicates (n = 4). Statistical significance was assessed using an unpaired Student’s t-test. (G, H) RT-qPCR analysis of intergenic sense eRNAs (E1-E3) (G) and the intragenic sense eRNA (E4) (H) transcribed from the *Tdp-43* enhancer in ESC and NPC states, presented as bar graphs (top) and line graphs (bottom). The expression level of the intragenic eRNA was normalized by dividing the cDNA synthesized with random hexamers by that synthesized with oligo(dT) primers. All RT-qPCR data are presented as mean ± SEM from three independent biological replicates (n = 3), except for Figure 4F. Statistical significance was assessed using an unpaired Student’s t-test.

Pluripotent stem cell differentiation involves the activation of lineage-specific gene programs alongside the repression of pluripotency and alternative lineage pathways, a process driven by dynamic and differential enhancer usage (Choukrallah et al. 2015). eRNAs are key regulators in this process, modulating gene expression in a cell – and tissue-type-specific manner throughout early development (Levine 2010; Bulger and Groudine 2011; Nord et al. 2013). To determine whether differential *Tdp-43* enhancer activity and eRNA expression occur in a context-dependent manner, we analyzed their dynamics during the transition from mESCs to NPCs. Differentiation was induced by EB formation followed by neural lineage induction (Figure 4(A)). As expected, pluripotency markers (*Pou5f1*, *Sox2*, *Nanog*, and *Klf4*) declined over time, confirming loss of stem cell identity (Figure 4(B)). Concurrently, lineage markers for ectoderm (*Nestin* and *Pax6*), endoderm (*Sox17*), and mesoderm (*T; Brachyury*) were upregulated (Figure 4(C)), indicating successful neural differentiation. Supporting these observations, immunofluorescence analysis revealed a marked increase in NESTIN protein expression in NPCs, consistent with its induction during differentiation (Figure 4(D)). Consistent with this, Western blotting provided complementary validation, confirming the same increase in NESTIN protein expression (Figure 4(E)).

To assess how *Tdp-43* expression changes during differentiation, we measured its transcript levels across ESC and NPC stages. *Tdp-43* expression progressively increased during this transition (Figure 4(F)), in line with its known roles in neural development, including mRNA stabilization and alternative splicing (Vogt et al. 2018; Baughn et al. 2023). Notably, distinct changes in eRNA expression were observed between mESCs and NPCs (Figure 4(G,H), S4A, and S4B). Intergenic eRNAs E1 and E3 were downregulated, whereas E2 was strongly induced (Figure 4(G)). Although antisense transcripts were generally less abundant than sense strands, E2 expression increased in both orientations (Fig. S4A). Notably, E2 activity appeared weaker in NPCs than in ESCs at the chromatin level, this likely reflects a gradual attenuation during differentiation rather than a complete loss of function (Figure 1(A), Figure 4(G,H), S4A, and S4B). In contrast, the intragenic enhancer eRNA E4 showed a moderate reduction in NPCs relative to ESCs, though its expression remained higher than E2 (Figure 4(H)). These patterns are consistent with our ChIP-seq data, which showed increased H3K27ac enrichment at E2 and E4 in NPCs but not in ESCs (Figure 1(A)). Our finding also aligns with a previous observation that sense-strand eRNAs from intergenic enhancers tend to have greater functional relevance than antisense strands (Schaukowitch et al. 2014) (Figure S4A-B). Together, these results suggest that differential enhancer usage occurs during neural lineage commitment, where E1 and E3 become inactivated while E2 and E4 are maintained or further activated. These dynamic regulatory changes likely contribute to the stage-specific upregulation of *Tdp-43* and its transcriptional fine-tuning.

Because enhancer activity can vary across developmental stages and tissue types (Heintzman et al. 2007; Creyghton et al. 2010), we next examined whether the *Tdp-43* enhancer regions identified in ESCs are also active in other tissues. To this end, we analyzed publicly available H3K27ac ChIP-seq datasets from mouse forebrain, heart, and liver across embryonic to postnatal stages. H3K27ac signals at E2 were weak or ambiguous in the forebrain, transiently enriched in the embryonic heart, and strongly activated in the liver during early postnatal stages. In contrast, E4 showed robust H3K27ac enrichment in the forebrain during embryonic and early postnatal stages, followed by a decline later in development, while remaining relatively stable in the heart and liver. Additionally, other enhancer regions (highlighted in yellow boxes) exhibited selective activation in the liver but not in the forebrain or heart (Figure S4C). These findings indicate that *Tdp-43* enhancer activity is both developmentally regulated and tissue-specific. In particular, E2 and E4 appear to be preferentially utilized during early neural differentiation, likely contributing to the spatial and temporal precision of *Tdp-43* expression essential for proper neural development. Based on these findings, we speculate that all four enhancers may contribute in ESCs, that NPCs exhibit a combination of relatively weak (E2) and strong (E4) enhancers, and that post-mitotic neurons in the forebrain rely predominantly on E4 enhancer activity to sustain *Tdp-43* expression.

### Differentiation-driven Tdp-43 eRNAs regulate neural lineage specification

**Figure 5.**
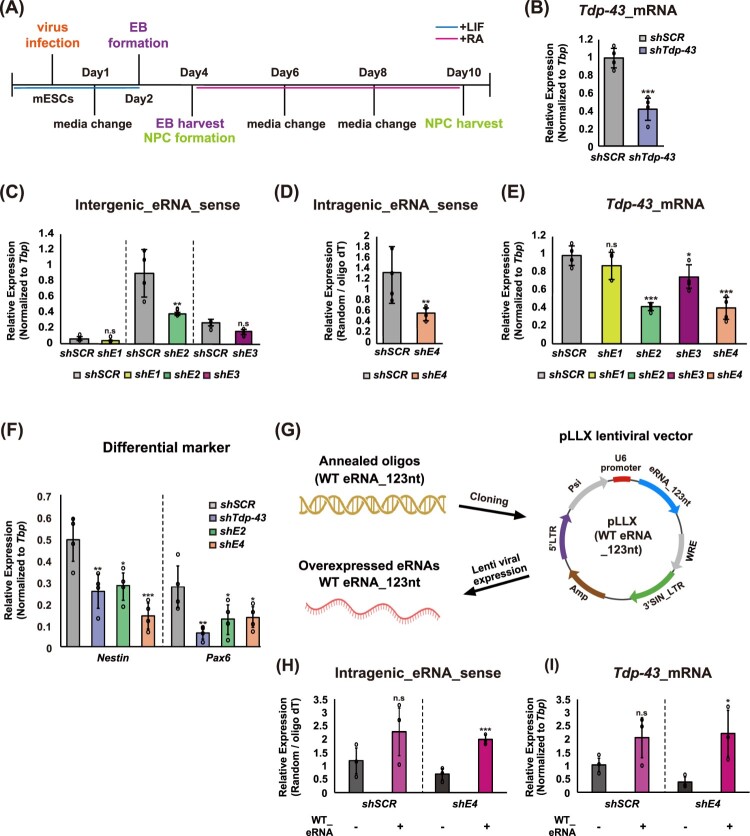
Functional requirement of retained eRNAs for *Tdp-43* expression and NPC lineage specification (A) Schematic diagram depicting lentiviral shRNA infection during the differentiation of ESCs into NPCs. (B) RT-qPCR analysis of *Tdp-43* mRNA expression in NPCs following shRNA-mediated knockdown of either *Tdp-43* or scrambled control. (C-E) RT-qPCR analysis of *Tdp-43* intergenic eRNAs (E1-E3, sense strand) (C), intragenic eRNA (E4, sense strand) (D), and *Tdp-43* mRNA expression (E) in NPCs following individual eRNA knockdown, compared to scrambled control. (F) RT-qPCR analysis of lineage-specific markers for ectoderm *(Nestin* and *Pax6*) following knockdown of *Tdp-43* mRNA, or intergenic (E2) and intragenic (E4) eRNAs, with the scrambled control included for comparison. (G) Schematic illustration of the WT eRNA construct design used for lentiviral rescue experiments. The diagram depicts the oligonucleotides used to generate the shRNA constructs for eRNA overexpression (WT eRNA_123 nt) and rescue experiments, along with the lentiviral vector backbone and shRNA expression cassette. (H, I) RT-qPCR analysis of *Tdp-43*_E4 eRNA (H) and *Tdp-43* mRNA (I) levels in ESCs following depletion of *Tdp-43*_E4 eRNA or scrambled control. After knockdown, cells were analyzed with or without infection of WT E4 eRNA rescue lentivirus prior to RNA extraction. The expression level of the intragenic eRNA was normalized by dividing the cDNA synthesized with random hexamers by that synthesized with oligo(dT) primers. RT-qPCR data are presented as mean ± SEM from three independent biological replicates (n = 3). Statistical significance was assessed using an unpaired Student’s t-test. All RT-qPCR data are presented as mean ± SEM from four independent biological replicates (n = 4), except for Figure 5H, I Statistical significance was assessed using an unpaired Student’s t-test.

Given that eRNA expression in ESCs directly promotes *Tdp-43* mRNA expression and supports pluripotency, we hypothesized that the context-specific differential enhancer usage during ESC-to-NPC differentiation contributes to neural lineage specification (Figure 1(A) and Figure 4(F–H)). To test this, we generated shRNA constructs targeting either *Tdp-43* mRNA or its associated eRNAs and induced neural differentiation under knockdown conditions (Figure 5(A)). Efficient knockdown was confirmed by a significant reduction in *Tdp-43* mRNA levels relative to the scrambled control (Figure 5(B)). We next assessed the functional contribution of individual *Tdp-43* eRNAs in NPCs by knocking down each of the four eRNAs: E1 and E3, which show relatively low expression, and E2 and E4, which are more abundantly expressed during differentiation (Figure 5(C–E)). Each knockdown effectively reduced the corresponding eRNA transcript, with the strongest depletion observed for E2 and E4. Consistently, *Tdp-43* mRNA levels were most significantly reduced following E2 and E4 knockdown, suggesting that these eRNAs play a predominant role in regulating *Tdp-43* expression at the NPC stage. To assess the impact of *Tdp-43* depletion in NPCs, we examined the expression of ectodermal markers (*Nestin* and *Pax6*) (Figure 5(F)). As expected, knockdown of *Tdp-43* led to a marked reduction in ectodermal marker expression. Similarly, knockdown of individual eRNAs also resulted in a general decrease in ectodermal gene expression (Figure 5(F)), with the most pronounced effect observed following E4 depletion. To further confirm the specificity of these effects, we examined potential off-target changes (Figure S5A-D). GRO-seq tracks revealed that *Masp2* is located adjacent to *Tdp-43* and partially overlaps with the E4 GRO-seq signal (Figure S5A). Consistent with this genomic proximity, both genes exhibited comparable expression levels in mESCs (Figure S5B). However, *Masp2* mRNA levels remained unchanged following shE4 knockdown (Figure S5C), indicating that the observed effects are not due to local spillover from the *Tdp-43* locus. Likewise, shE4 treatment did not affect the expression of *Nsun2* mRNA (chr4), which is located on a different chromosome and was used as an independent off-target reference (Figure S5D). These results further support the specificity of the E4 eRNA knockdown. Consistent with an earlier report (Schaukowitch et al. 2014), the antisense strand of the intergenic eRNA exhibited limited functional activity compared with its sense counterpart (Figure S6A-C).

To further verify that the observed phenotype was specifically attributable to enhancer loss rather than promoter-proximal transcriptional spillover, we conducted a rescue experiment focusing on the E4 enhancer, which is located in close proximity to the *Tdp-43* transcription start site (TSS). Although knockdown of both E2 and E4 reduced *Tdp-43* expression, rescue analysis was performed specifically for E4 to determine whether the transcription observed at this locus reflects promoter-proximal pausing or transcriptional spillover, or instead represents genuine enhancer activity. For this purpose, we generated a lentiviral construct expressing the WT E4-derived eRNA and confirmed that WT eRNA overexpression alone increased both E4 eRNA and *Tdp-43* mRNA levels, indicating that the system effectively recapitulates enhancer-associated eRNA function. Importantly, restoration of WT E4 eRNA expression after shE4 treatment successfully reversed the reduction in *Tdp-43* mRNA (Figure 5(G-I)), thereby establishing a causal and RNA-dependent role for E4 in regulating *Tdp-43* transcription. Together, these findings demonstrate that E4-derived transcription reflects bona fide enhancer activity rather than promoter-proximal transcriptional noise. They further indicate that differentiation-driven enhancer dynamics result in the selective retention of functionally relevant *Tdp-43* eRNAs. These eRNAs continuously sustain *Tdp-43* transcription and, in turn, enable TDP-43 to execute its transcriptional regulatory roles as a key mediator of neural progenitor differentiation and lineage commitment.

## Discussion

TDP-43 is a DNA/RNA-binding protein that plays essential roles in RNA metabolism, including transcription, splicing, transport, and stability, and is indispensable for neural development. Dysregulation of TDP-43 has been strongly implicated in various neurodegenerative diseases such as ALS and FTLD. While TDP-43 has been predominantly studied in the context of post-transcriptional and especially post-translational regulation, our study uncovers an additional and previously underexplored complementary layer of transcriptional control mediated by enhancer RNAs (eRNAs), acting in a cell state-dependent manner during early neural differentiation. We focused on neural progenitor cells (NPCs) because the transition from pluripotency to neural lineage commitment represents a key developmental window characterized by extensive enhancer reprogramming. Whether the identified eRNAs, particularly E2 and E4, persist or retain regulatory function in post-mitotic neurons remains to be determined; nevertheless, this question represents an important avenue for future investigation. Notably, H3K27ac ChIP-seq profiles from mouse forebrain across multiple developmental stages (E11.5 to P56) revealed sustained enrichment at the E2 and E4 loci (Figure S4C), suggesting that these enhancers remain active from embryonic through postnatal stages, potentially extending into mature neuronal contexts. Given the well-established association between TDP-43 dysregulation and neurodegenerative diseases such as ALS, determining whether enhancer-mediated transcriptional regulation is preserved in mature neuronal subtypes may provide valuable insight into disease vulnerability and stage-specific enhancer utilization. Through integrative epigenomic and transcriptomic analyses, we identified multiple enhancer regions within the *Tdp-43* locus in mouse embryonic stem cells (mESCs), including both intergenic and intragenic enhancers, that transcribe distinct eRNAs. These eRNAs exhibit dynamic, region-specific expression changes during ESC-to-NPC differentiation, suggesting that enhancer activity is selectively remodeled during lineage commitment (Figures 4 and 5). These changes in enhancer usage aligns with developmental stage-specific regulatory needs (Figure 1(A), Figure 4(F–H), S4C). The selectively retained eRNAs promote *Tdp-43* transcription, enabling TDP-43 to exert its transcriptional regulatory function in both maintaining pluripotency and directing neural differentiation. Knockdown of individual eRNAs, particularly E2 and E4 which are highly expressed in NPCs, led to a significant reduction in *Tdp-43* mRNA levels and disrupted the expression of pluripotency and lineage markers, with the strongest effects observed in ectodermal genes. These results indicate that *Tdp-43* eRNAs function as integral, context-dependent regulators of *Tdp-43* expression and downstream developmental programs.

Intergenic enhancers are located outside gene bodies and typically regulate the expression of distant genes. In contrast, intragenic enhancers reside within gene loci and can regulate both their host genes (Kim et al. 2010; Kowalczyk et al. 2012; Bressin et al. 2023). Although both types of enhancers engage with transcription factors and contribute to the regulation of gene expression, they differ substantially in their genomic context and regulatory mechanisms (Kim et al. 2010; Calo and Wysocka 2013; Schaukowitch et al. 2014; Shlyueva et al. 2014; Heinz et al. 2015; Kim and Shiekhattar 2015). While previous studies have primarily focused on intergenic enhancers and their role in facilitating transcription through promoter-enhancer looping and chromatin organization (Kim and Shiekhattar 2015), intragenic enhancers have received less attention. Nevertheless, intragenic elements have been implicated in regulating alternative promoter usage and modulating the transcriptional activity of their host genes (Kowalczyk et al. 2012; Cinghu et al. 2017; Bressin et al. 2023). However, the prevalence and functional roles of intragenic enhancers in pluripotent stem cells remain insufficiently explored. Our analysis revealed that approximately 40-50% of all putative enhancers in mESCs are located within gene bodies (Figure 1(B)), which is consistent with prior reports (Heintzman et al. 2009; Borsari et al. 2021). Moreover, these enhancer regions were marked by active histone modifications and displayed chromatin signatures correlated with cell-type-specific gene expression, highlighting their potential roles in maintaining cellular identity and guiding developmental transitions.

During the transition from ESCs to NPCs, we observed differential enhancer activity at the *Tdp-43* locus accompanied by distinct changes in eRNA expression (Figure 1(A), Figure 4(F–H)). eRNAs transcribed from E1 and E3 were downregulated upon differentiation, whereas E2–and E4-derived eRNAs were maintained or upregulated in NPCs, coinciding with increased H3K27ac enrichment at these loci (Figure 1(A)). Although chromatin-based signals at E2 appeared weaker in NPCs than in ESCs, RT-qPCR analysis revealed increased E2 eRNA expression in NPCs (Figure 1(A), Figure 4(G)), indicating that E2 remains transcriptionally active during the ESC-to-NPC transition. Together with tissue– and stage-resolved H3K27ac profiles showing context-dependent activation of E2 and E4 across forebrain and liver tissues (Fig. S4C), these findings suggest that enhancer usage at the *Tdp-43* locus is dynamically tuned across developmental contexts rather than being simply gained or lost. Consistent with this model, functional knockdown of *Tdp-43* eRNAs resulted in a significant reduction of *Tdp-43* mRNA levels in both ESCs and NPCs (Figure 3(G–K), Figure 5(E,F)), underscoring the essential contribution of enhancer-derived eRNAs to context-dependent regulation of *Tdp-43* expression.

To directly assess the function of individual eRNAs, we performed knockdown experiments targeting each of the four eRNAs. Suppressing individual eRNAs led to significant reductions in *Tdp-43* mRNA levels in both ESCs and NPCs (Figure 3(E–G), Figure 5(C–E)), suggesting that these eRNAs act as integral components of *Tdp-43* transcriptional control in a context-dependent manner. Notably, knockdown of E2 and E4, which are the most abundantly expressed eRNAs in NPCs, resulted in the greatest reduction of *Tdp-43* expression and downregulation of ectodermal lineage markers (Figure 5(E,F)). These effects mirrored those observed following direct *Tdp-43* knockdown, indicating that the retained eRNAs actively contribute to sustaining *Tdp-43* expression during early neural differentiation, which may in turn modulate lineage marker expression (Figure 5(B–F)). The partial discrepancy between *Tdp-43* mRNA reduction (shE2) and lineage marker changes (shE4) likely reflects secondary or indirect effects, such as *Tdp-43* dosage sensitivity and network-level buffering, rather than off-target activity (Chalancon et al. 2012; Lee et al. 2016; Yang et al. 2021). While eRNAs could in principle exert broader regulatory influences, current evidence supports their predominant *cis*-acting functions. We also note that global transcriptomic profiling after eRNA knockdown would provide deeper insights into such broader effects, but this was beyond the scope of the present study and represents an important direction for future work. Furthermore, *Tdp-43* depletion reduced the expression of pluripotency markers in ESCs and impaired the expression of germ layer-specific genes in NPCs, with the strongest effect observed in ectodermal markers (Figure 3(G–K), Figure 5(E,F)). Together, these results suggest that distinct subsets of *Tdp-43* eRNAs exhibit dynamic expression changes and function at different developmental stages, enhancing *Tdp-43* transcription and influencing its regulatory activity in a context-dependent manner, thereby fine-tuning transcriptional output during neural lineage commitment.

**Figure 6.**
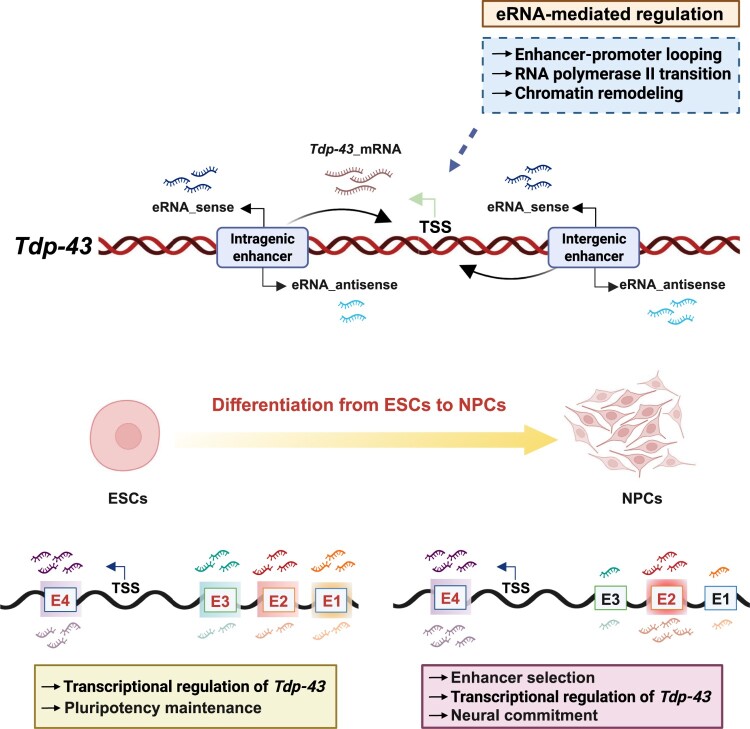
Model of eRNA-mediated transcriptional regulation of *Tdp-43* during ESC-to-NPC differentiation. Schematic model illustrating how intergenic and intragenic eRNAs dynamically regulate *Tdp-43* transcription during ESC-to-NPC differentiation. General eRNA-mediated regulatory concepts, including enhancer-promoter communication and transcriptional modulation, are schematically illustrated based on prior studies (Kim and Shiekhattar 2015), with broken boxed elements indicating inferred interactions. In ESCs, a subset of eRNAs promotes *Tdp-43* expression to maintain pluripotency. During neural commitment, enhancer activity is reconfigured, leading to the induction of distinct eRNAs that sustain *Tdp-43* expression in NPCs. This shift reflects changes in enhancer usage and stage-specific eRNA regulation that continuously modulate *Tdp-43* transcription in NPCs. Knockdown of these eRNAs reduces *Tdp-43* levels and disrupts both pluripotency and neural differentiation. This model highlights stage-specific eRNA regulation as a key mechanism for fine-tuning *Tdp-43* function during early neural development.

Our findings demonstrate that eRNAs transcribed from both intergenic and intragenic enhancer regions of the *Tdp-43* locus function cooperatively to regulate its expression in a temporally and spatially controlled manner. This combinatorial and temporally coordinated regulation constitutes a novel mechanism of transcriptional control during early neural development (Figure 6). Furthermore, our findings raise broader implications for understanding how disruptions in enhancer function may contribute to TDP-43-related neurodevelopmental and neurodegenerative processes. Dysregulation of TDP-43 is a hallmark of ALS and FTLD, disorders in which enhancer dysfunction and transcriptional misregulation are increasingly recognized. The observation that eRNAs modulate *Tdp-43* transcription raises the possibility that impaired enhancer function may drive aberrant TDP-43 expression or pathological accumulation in these conditions. Finally, our results suggest a tight coupling between the transcriptional and post-transcriptional regulatory roles of TDP-43. By influencing *Tdp-43* transcriptional output, eRNAs may affect the intracellular levels of TDP-43 protein and thereby modulate downstream RNA processing events in which TDP-43 is involved. These findings establish a new framework for exploring enhancer RNA biology in the context of neurodevelopment and neurodegeneration, and highlight eRNAs as promising candidates for biomarker discovery or therapeutic intervention in TDP-43-associated diseases, while complementing rather than replacing the well-established post-translational regulation of TDP-43.

## Supplementary Material

Jang_TDP43_Suppl.pdf

Jang_TDP43_Suppl_Table_1-5.xlsx
