# Supplementary material for: Enhancer RNA-mediated transcriptional regulation of TDP-43 during early neural lineage specification
Source: Anim Cells Syst (Seoul). 2026 Mar 26;30(1):320–41. doi: 10.1080/19768354.2026.2643998 (PMC13023014; doi:10.1080/19768354.2026.2643998)
Supplement: Jang_TDP43_Suppl.pdf [file TACS_A_2643998_SM7928.pdf]

Enhancer RNA-mediated transcriptional regulation of TDP-43 during early neural lineage specification

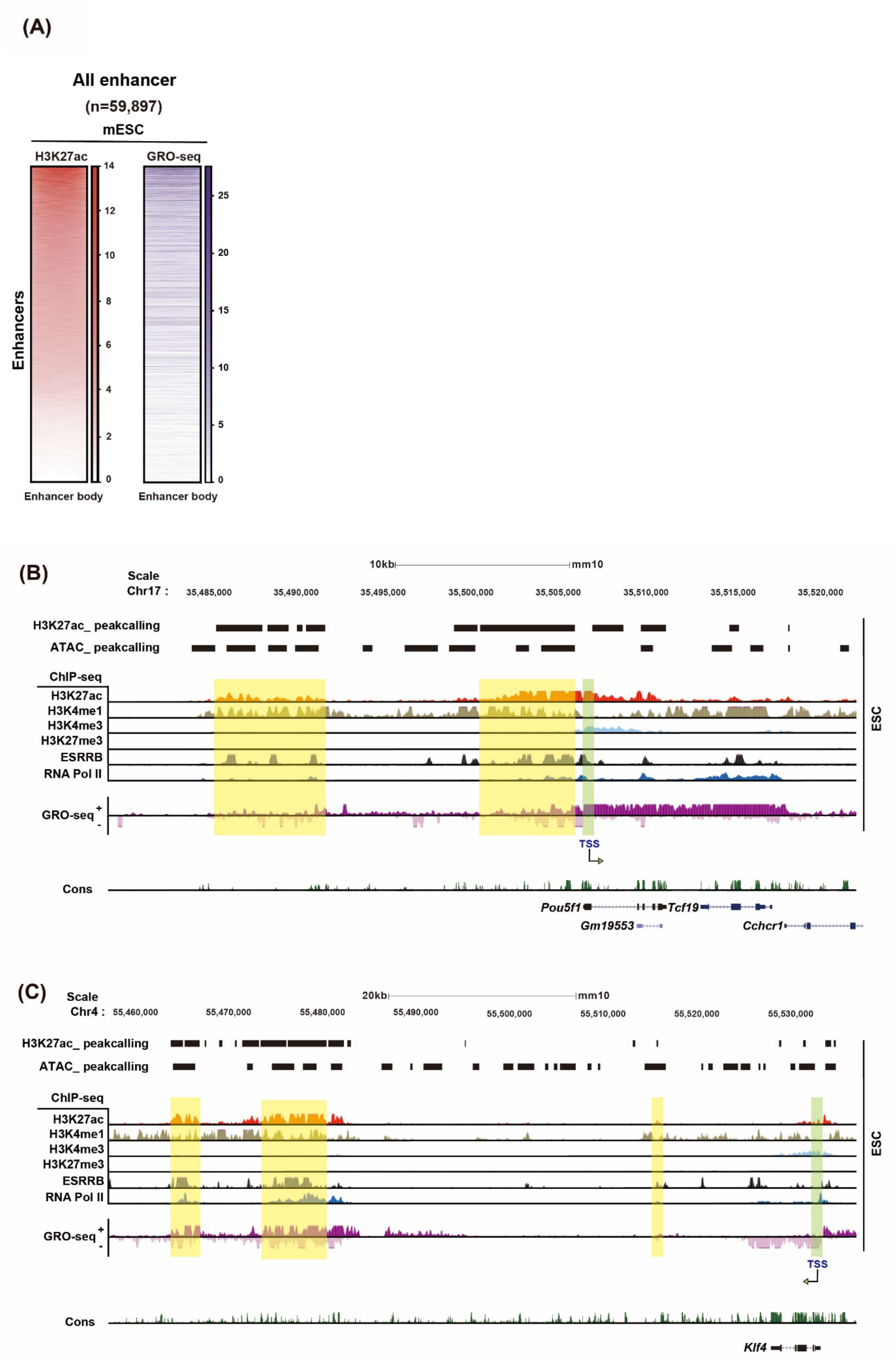

(A)
All enhancer
(n=59,897)
mESC
H3K27ac
GRO-seq
Enhancers
Enhancer body
Enhancer body
(B)
Scale
Chr17 :
10kb
mm10
35,485,000
35,490,000
35,495,000
35,500,000
35,505,000
35,510,000
35,515,000
35,520,000
H3K27ac_ peakcalling
ATAC_ peakcalling
ChIP-seq
H3K27ac
H3K4me1
H3K4me3
H3K27me3
ESRRB
RNA Pol II
GRO-seq
ESC
TSS
Cons
Pou5f1
Tcf19
Gm19553
Cchcr1
(C)
Scale
Chr4 :
20kb
mm10
55,460,000
55,470,000
55,480,000
55,490,000
55,500,000
55,510,000
55,520,000
55,530,000
H3K27ac_ peakcalling
ATAC_ peakcalling
ChIP-seq
H3K27ac
H3K4me1
H3K4me3
H3K27me3
ESRRB
RNA Pol II
GRO-seq
ESC
TSS
Cons
Klf4

Fig. S1. Genome-wide and locus-specific validation of enhancer-associated chromatin features in mESCs

(A) Heatmaps showing the genome-wide correlation between H3K27ac ChIP-seq and GRO-seq signal intensities across all putative enhancer regions (n = 59,897) in mESCs. (B, C) UCSC Genome Browser views of the *Pou5f1* (B) and *Klf4* (C) loci used as positive controls for ChIP-seq and ATAC-seq data quality assessment. Browser tracks display histone modification profiles (H3K27ac, H3K4me1, H3K4me3, H3K27me3), transcription factor binding (ESRRB), RNA polymerase II occupancy, and strand-specific nascent transcription (GRO-seq; upper = plus, lower = minus strand). "Cons" denotes PhastCons conservation scores across vertebrate species based on the mm10 genome alignment. Peak calling results for H3K27ac and chromatin accessibility (ATAC-seq) are shown above each track. Well-characterized enhancer regions near *Pou5f1* and *Klf4* are highlighted in yellow boxes. These loci exhibit strong enrichment of canonical enhancer-associated chromatin marks (H3K27ac and H3K4me1), high chromatin accessibility, RNA polymerase II binding, and robust bidirectional nascent transcription, validating the technical quality and dynamic range of the epigenomic datasets used in this study.

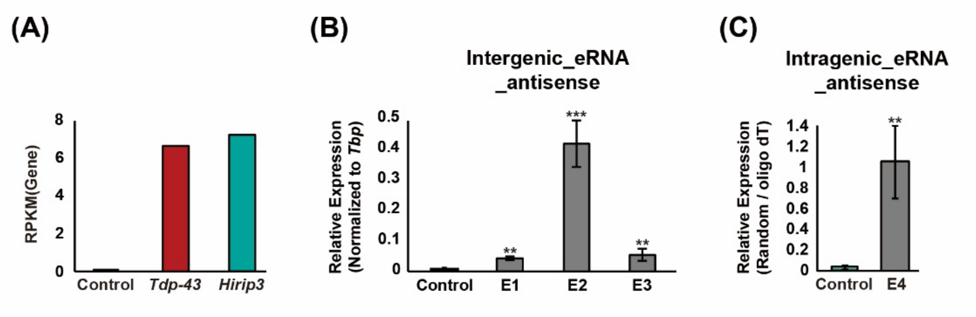

Fig. S2 Antisense strand expression of *Tdp-43* eRNAs in mESCs

(A) RPKM values comparing control, *Tdp-43,* and the *Hirip3* (RPKM=7.02529) in ESCs. (B) RT-qPCR analysis comparing the antisense strand expression of *Tdp-43* intergenic enhancers (E1-E3) to the intergenic region of another gene with similar overall expression, *Hirip3* (Control). (C) RT-qPCR analysis comparing the antisense strand expression of the *Tdp-43* intragenic enhancer (E4) to a non-enhancer intragenic region within the *Tdp-43* gene body (Control). The expression level of the intragenic eRNA was normalized by dividing the cDNA synthesized with random hexamers by that synthesized with oligo(dT) primers. All RT-qPCR data are presented as mean ± SEM from three independent biological replicates (n = 3). Statistical significance was assessed using an unpaired Student's t-test.

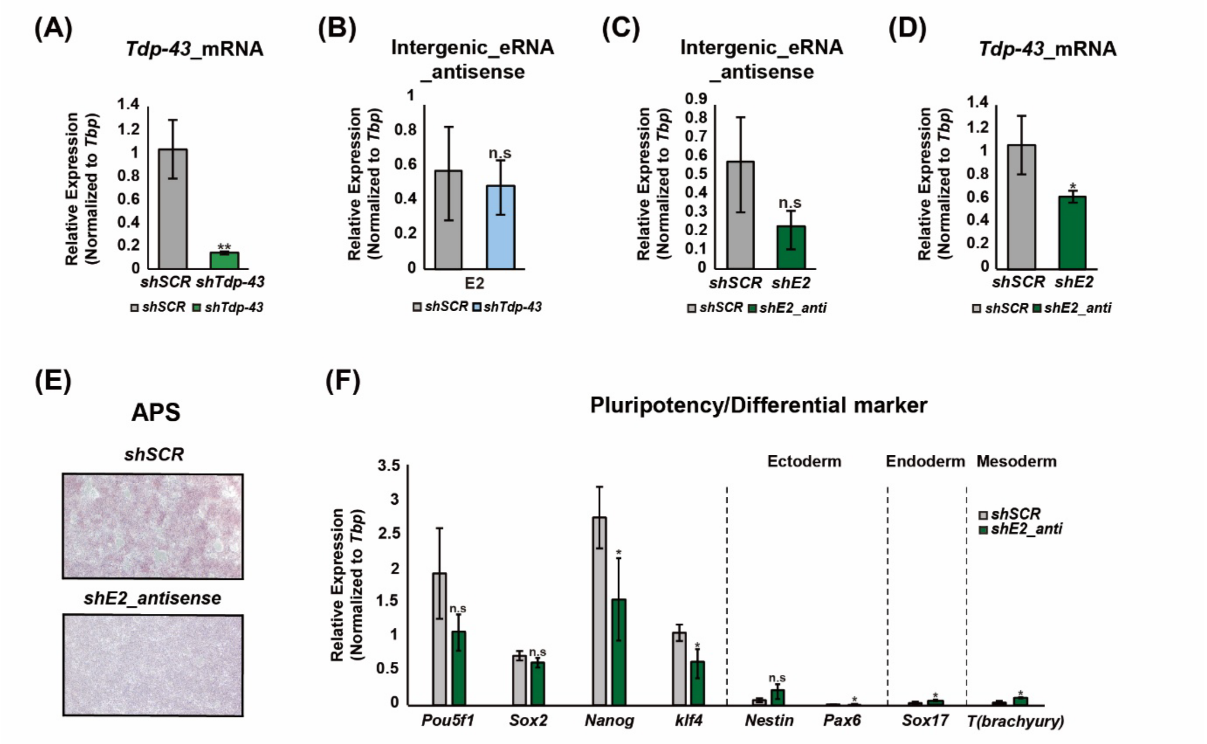

Fig. S3 Depletion of antisense eRNA expression from a *Tdp-43* enhancer in mESCs

(A) RT-qPCR analysis of *Tdp-43* mRNA expression in mESCs following shRNA-mediated knockdown of either *Tdp-43* or scrambled control. The same knockdown dataset shown in Figure 3A is reused here for consistency and to provide a comprehensive view of the effects across different conditions, including eRNA knockdown. (B) RT-qPCR analysis of intergenic antisense eRNA (E2) expression from the *Tdp-43* enhancer following knockdown of *Tdp-43* or scrambled control. (C) RT-qPCR analysis of intergenic antisense eRNA (E2) expression from the *Tdp-43* enhancer following knockdown of eRNA compared to the scrambled control. (D) RT-qPCR analysis of *Tdp-43* mRNA expression following knockdown of eRNA compared to the scrambled control. (E) AP staining of mESCs following knockdown of scrambled control and antisense eRNA (E2). (F) RT-qPCR analysis of pluripotency (*Pou5f1*, *Sox2*, *Nanog, a*nd *Klf4*) and lineage-specific markers for ectoderm (*Nestin* and *Pax6*), endoderm (*Sox17*), and mesoderm (*T; Brachyury*) following knockdown of scrambled control and antisense eRNA (E2). All RT-qPCR data are presented as mean ± SEM from three independent biological replicates (n = 3). Statistical significance was assessed using an unpaired Student's t-test.

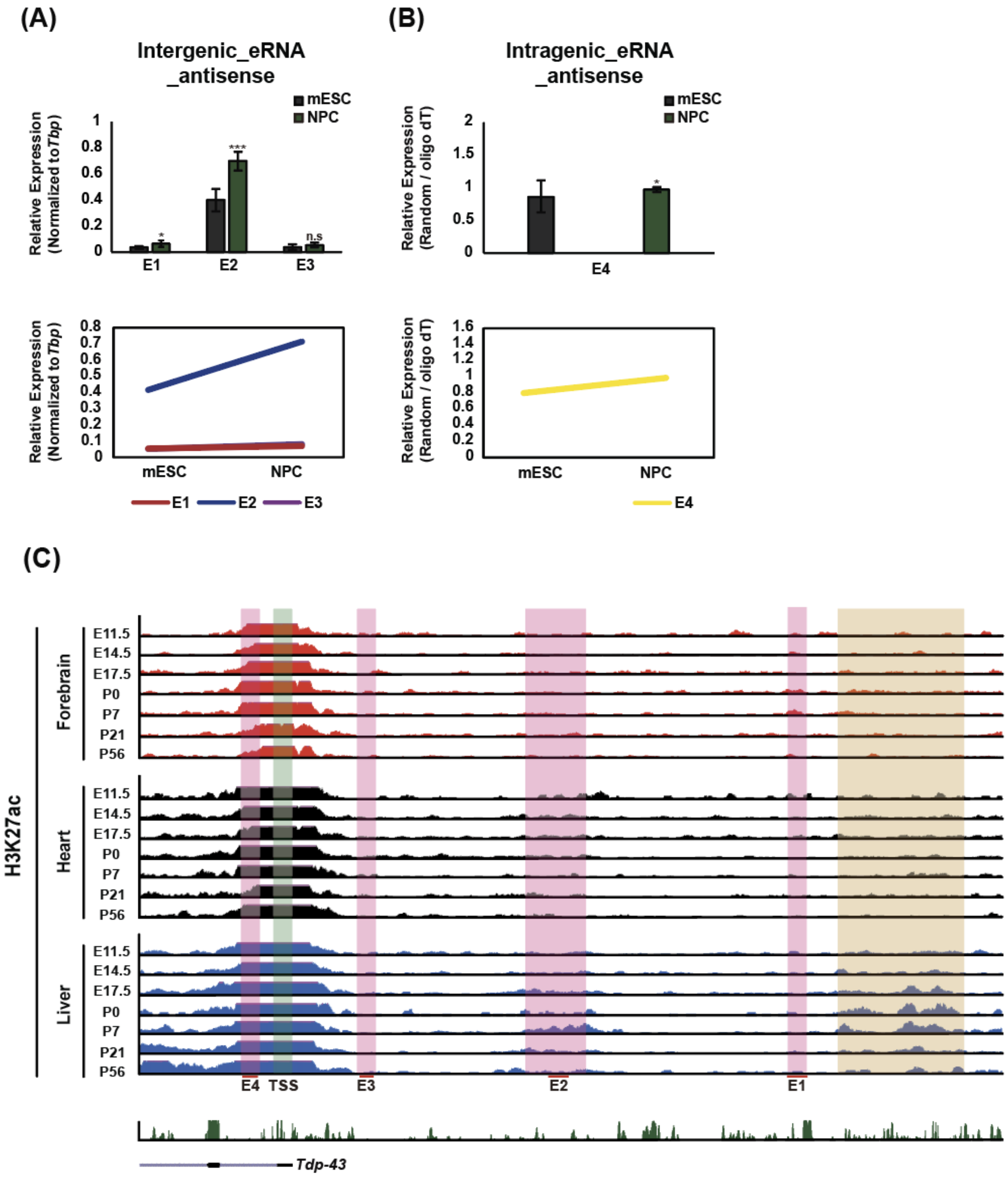

(A)
Intergenic_eRNA _antisense
mESC
NPC
Relative Expression (Normalized to Tbp)
E1
E2
E3
n.s
mESC
NPC
E1
E2
E3
(B)
Intragenic_eRNA _antisense
mESC
NPC
Relative Expression (Random / oligo dT)
E4
mESC
NPC
E4
(C)
H3K27ac
Forebrain
Heart
Liver
E11.5
E14.5
E17.5
P0
P7
P21
P56
E4
TSS
E3
E2
E1
Tdp-43

Fig. S4 Antisense eRNA expression and H3K27ac ChIP-seq profiles at the *Tdp-43* locus during ESC-to-NPC differentiation or tissue development

(A, B) RT-qPCR analysis of intergenic antisense eRNAs (E1-E3) (A) and intragenic antisense eRNA (E4) (B) expression from the *Tdp-43* enhancer in ESC and NPC states, shown as bar graphs (top) and line graphs (bottom). The expression level of the intragenic eRNA was normalized by dividing the cDNA synthesized with random hexamers by that synthesized with oligo(dT) primers. All RT-qPCR data are presented as mean ± SEM from three independent biological replicates (n = 3). Statistical significance was assessed using an unpaired Student's t-test. (C) UCSC Genome Browser view of the *Tdp-43* locus, displaying H3K27ac ChIP-seq data from forebrain, heart, and liver tissues across developmental stages. The TSS is highlighted in green, the ESC-specific enhancer region is marked in pink, and a region showing a liver-specific increase in H3K27ac signal during differentiation is denoted in orange.

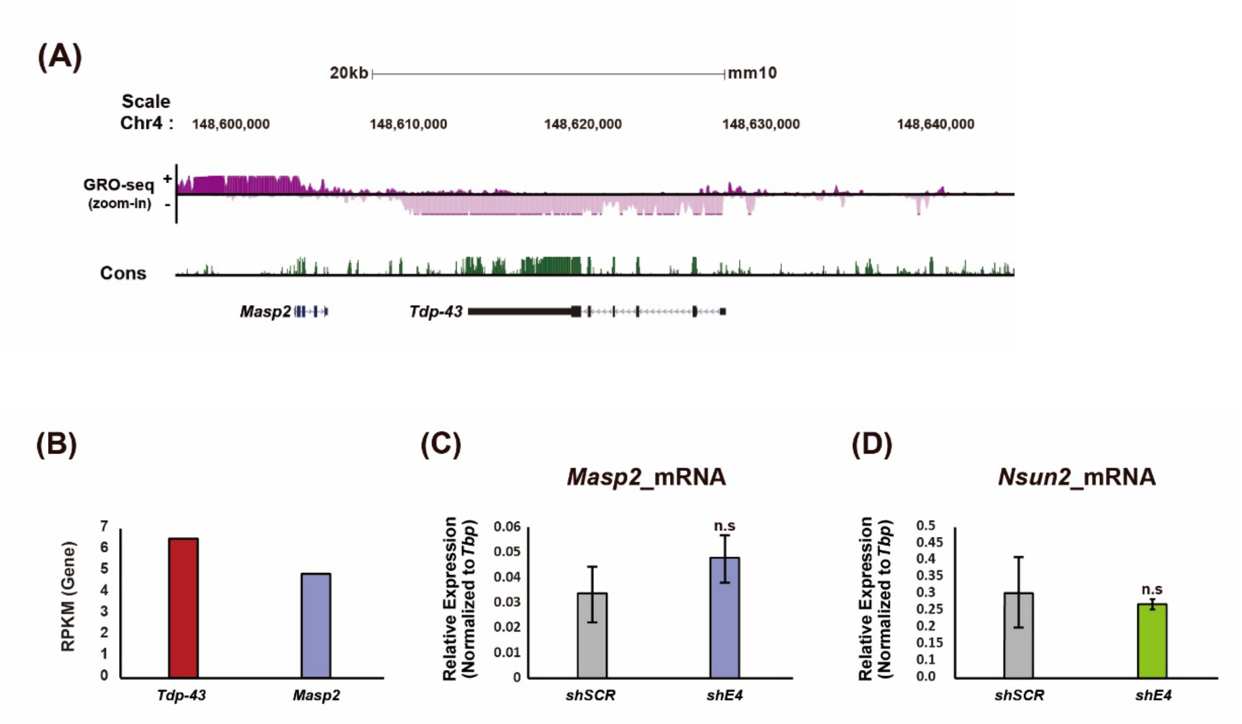

Fig. S5 *Tdp-43* enhancer activity and off-target analysis of *Masp2* expression

(A) GRO-seq tracks (strand-specific) in mESCs showing the transcriptional landscape at the *Tdp-43* locus and the neighboring gene *Masp2*. Indicate transcription from the plus and minus strands, respectively. (B) RPKM values of *Tdp-43* and *Masp2* (RPKM=4.86988) in mESCs. (C) Relative mRNA expression of the neighboring gene *Masp2* following shRNA-mediated knockdown of the E4 enhancer RNA (shE4) compared with scrambled control (shSCR*)*. (D) Relative mRNA expression of *Nsun2*, a gene located outside the E4 enhancer region but known to play a critical role in maintaining mESC pluripotency and during cellular differentiation. Expression levels were examined following shRNA-mediated knockdown of E4 enhancer RNA (shE4) compared with scrambled control (shSCR). No significant change was observed, indicating the specificity of the eRNA knockdown. All RT-qPCR data are presented as mean ± SEM from three independent biological replicates (n = 3). Statistical significance was assessed using an unpaired Student's t-test.

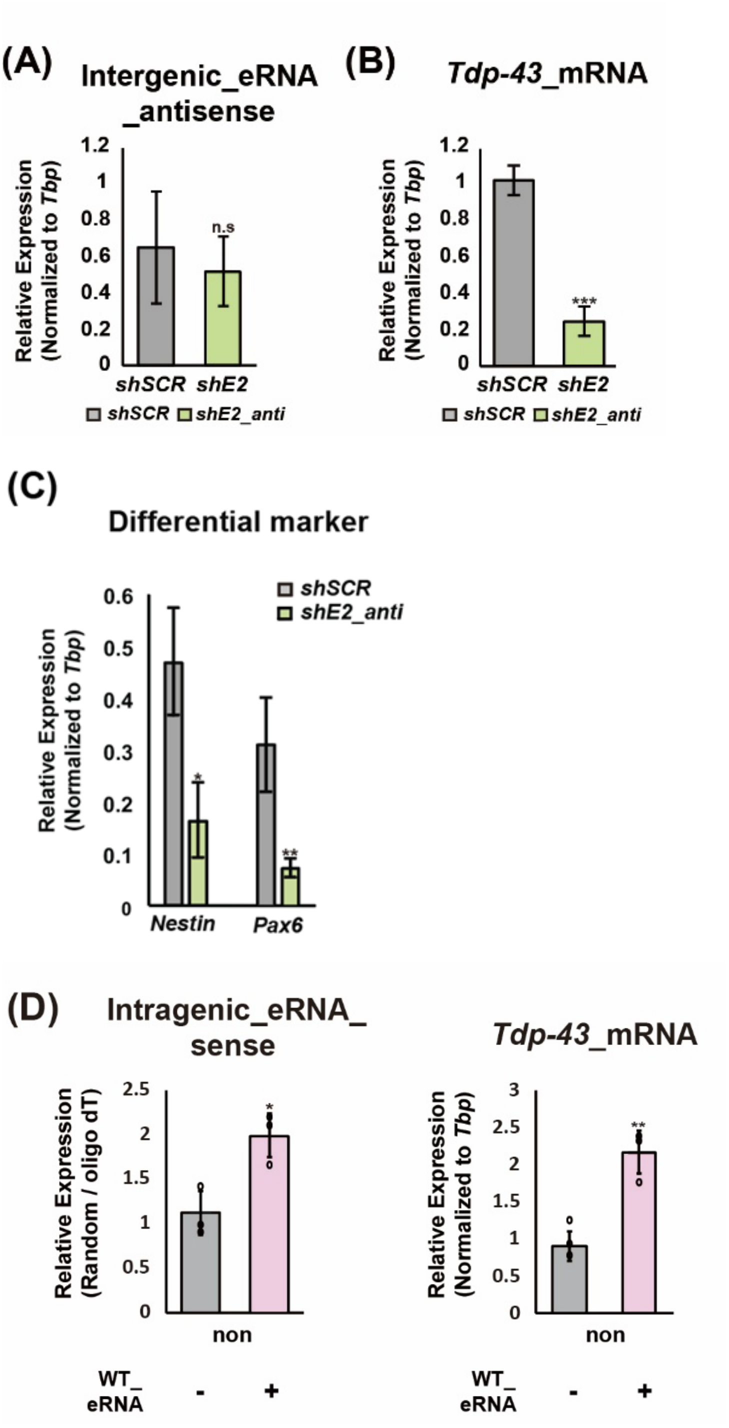

Fig. S6 Effects of antisense eRNA knockdown on *Tdp-43* and lineage gene expression in NPCs

(A, B) RT-qPCR analysis of intergenic antisense eRNA (E2) expression from the *Tdp-43* enhancer (A), as well as *Tdp-43* mRNA expression (B) in NPCs following knockdown of eRNA compared to the scrambled control. (C) RT-qPCR analysis of lineage-specific markers for ectoderm (*Nestin* and *Pax6*) following knockdown of scrambled control and antisense eRNA

(E2). (D) RT-qPCR analysis of *Tdp-43*_E4 eRNA and *Tdp-43* mRNA expression in wild-type ESCs transduced with 1,500 μl of WT E4 eRNA rescue lentivirus in the absence of shRNA-mediated knockdown. Cells were harvested 4 days post-infection for RNA analysis. All RT-qPCR data are presented as mean ± SEM from three independent biological replicates (n = 3). Statistical significance was assessed using an unpaired Student's t-test.
